# Variable clinical presentation of split hand/foot malformation syndrome in a family with microduplication of 10q24.32: a case report

**DOI:** 10.3389/fgene.2023.1303807

**Published:** 2024-01-05

**Authors:** Daria Akimova, Tatiana Markova, Maria Ampleeva, Mikhail Skoblov

**Affiliations:** ^1^ Research Centre for Medical Genetics, Moscow, Russia; ^2^ Independent Clinical Bioinformatics Laboratory, Moscow, Russia

**Keywords:** split hand/foot malformation, whole genome sequencing, mosaicism, pathogenic duplications, ectrodactyly

## Abstract

SHFM (Split Hand/Foot Malformation) is a heterogeneous group of disorders characterized by the presence of clefts in the hands and feet, along with syndactyly of the digits. In this article, we describe a family in which two members exhibit characteristic developmental abnormalities associated with SHFM, presenting with variable clinical features. Using whole-genome sequencing, we identified a microduplication of a chromosomal segment on locus 10q24.32, specifically spanning positions 102934495 to 103496555, encompassing genes *BTRC*, *POLL*, *FBXW4* and *LBX1* in the proband. Genomic duplications, including these genes, were previously described in patients diagnosed with the third type of SHFM. We validated the presence of this structural rearrangement in 7 family members, including the proband and the proband’s father. Remarkably, further investigation demonstrated that the detected duplication exhibits a mosaic state in the phenotypically normal paternal grandmother of the proband, thereby providing a plausible explanation for the absence of a pathological phenotype in her.

## 1 Introduction

Split hand/foot malformation (SHFM) is a rare genetic disorder that affects the development of the limbs, resulting in underdevelopment (hypoplasia) of the central digital rays, variable fusion of the remaining fingers and feet, or a deep median cleft of the hand and/or foot (Elliott et al., 2005). Also known as ectrodactyly, this condition can vary in severity, ranging from a minor cosmetic issue to a significant disability. According to orphanet database of rare diseases the prevalence of SHFM is approximately 5 per 100,000 births (Rath, 2022). SHFM accounts for 15% of all limb reduction defects (Holder-Espinasse et al., 2019). The condition can be inherited in an autosomal dominant, autosomal recessive, or X-linked manner. While most cases described in the literature are familial forms, sporadic cases also occur.

Split-Hand/Foot Malformation results from abnormalities in multiple genomic locations and includes 6 types of disease (Sowinska-Seidler et al., 2014). The most common type of Split hand/foot malformation is the third type (Guero and Holder-Espinasse, 2019). It is caused by a duplication of a segment of chromosome 10. SHFM type 3 was first described in two siblings in 1987, showing symmetric severe distal limb deficiencies affecting all limbs, along with microretrognathia and microstomia (Buttiens and Fryns, 1987). The size of the detected duplication varies from 120 kb, including only the *BTRC* gene (Qiu et al., 2022), to 658 kb, including the genes *TLX1, LBX1, BTRC, POLL, DPCD,* and *FBXW4* (Li et al., 2015; Lai et al., 2020). As of now, no critical region responsible for the SHFM type 3 phenotype has been identified, but there is evidence suggesting that a region within exon 1 of the *BTRC* gene may play a crucial role in causing the SHFM type 3 phenotype by acting as a regulatory element (Dai et al., 2013) Furthermore, recent studies have shown that the pathological duplication underlying the pathogenesis of SHFM type 3 leads to chromatin restructuring, subsequently resulting in the ectopic activation of the *Lbx1* and *BTRC* genes in the apical ectodermal ridge (AER). This mechanism is induced by *Fgf8* AER enhancers. Given cases where the duplication includes only *BTRC* alone, it has been hypothesized that the abnormal expression of *BTRC* could be the primary factor contributing to the pathology of the human SHFM3 phenotype (Cova et al., 2023).

Based on several dozens of patients with a molecularly confirmed diagnosis of SHFM type 3, it can be concluded that there is genetic heterogeneity and high clinical variability of the disease, even among members of the same family (Duijf et al., 2003; Elliott and Evans, 2006; Dai et al., 2013). Some cases have been described as mosaic states of duplication, where a milder course of the disease or even the complete absence of the pathological phenotype was observed (Auerbach, 1956; De Smet et al., 2001; Dimitrov et al., 2010; Filho et al., 2011).

Members of the family described in our study, despite having the same structural rearrangement, exhibit varying degrees of clinical manifestations of the disease - ranging from mild, almost asymptomatic, to extremely severe.

## 2 Materials and methods

### 2.1 Subjects

The proband, 6 month old boy, and his parents underwent a detailed clinical examination and genetic investigation at the Research Center for Medical Genetics, Moscow, Russia.

All research participants gave informed consent for the clinical examination and publication of their anonymized data. The study was performed in accordance with the Declaration of Helsinki and approved by the Institutional Review Board of the Research Center for Medical Genetics., Moscow, Russia.

### 2.2 Genome sequencing and variants calling

The whole genome sequencing of the proband’s DNA sample, obtained from peripheral blood, was conducted in-house. DNA extraction from whole blood was carried out using the Quick-DNA Miniprep Kit (Zymo Research, California, United States), following the manufacturer’s protocol. To assess DNA purity, absorbance measurements were taken at both 260/280 nm and 230/260 nm using a DS-11 FX + spectrophotometer/fluorometer (DeNovix, Wilmington, United States).

Library preparation (PCR-Free) was performed using MGI platforms following their respective protocols. Subsequently, paired-end sequencing (2 × 150) was executed on the DNBSEQ-T7 platform from MGI. The data processing was carried out using “NGSData-Genome” program (Beskorovainy N.S. Program “NGSData”//Certificate of NGSData-Genome”//Certificate of State Registration of Computer Programs No. 2021662119.2021.) The reads were aligned to the reference genome hg19 using bwa v.0.7.17-r1188. Variants calling was performed with strelka2 v.2.9.10 and gatk v.4 algorithms. Calling of copy number variations were assessed using the cnvkit 0.9.9, while structural variants were detected with Manta v.1.6.0. Additionally, tandem repeats were analyzed using ExpansionHunterDenovo. Variant annotation–SnpEff v5.0, annovar v.2017, vep v.104.3. Splice predictors–dbNSFP v.4, SPiP v.2.1, mmsplice v.2.3, spliceai v.1.3.1, spidex v.1.

### 2.3 Segregation studies

Validation of the obtained results and segregation analysis were conducted using PCR with two primer pairs designed to amplify the reference locus (5′-AAC​AAA​ATC​AAG​AGA​GCC​AAA​GA-3′, 5′-GGC​CAG​TAA​TTT​ACC​CAA​GG-3′) and the locus at the border of the reference and duplicated regions (5′-TGC​CAC​CCC​CAC​TAT​TTT​AC-3′, 5′-TTC​TTC​TAG​GAA​ATA​ATG​GAG​AAT​GTT-3′). The DNA samples from the proband’s peripheral blood, his parents, paternal grandmother, grandfather, great-grandmother, and paternal aunt were used as templates. The PCR was conducted using ProFlex PCR System (Applied Biosystems, California, United States). The visualization of the PCR products obtained was carried out using agarose gel electrophoresis. The level of mosaic deletion in the proband’s paternal grandmother’s blood sample was assessed densitometrically.

## 3 Results

The boy was born after 42 weeks of gestation from the first pregnancy a child from the first operative delivery through a cesarean section in a non-consanguineous family. The delivery was normal with an APGAR score of 7/8. At birth, the baby weighed 3,190 g and measured 50 cm in length. He achieved all early motor milestones successfully.

**FIGURE 1:**
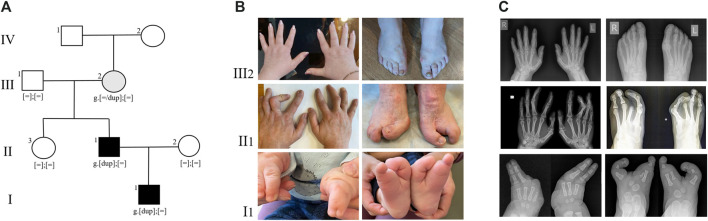
**(A)** Pedigree of the SHFM type 3 family. Family members: I1—proband, II1- proband’s father, II2- proband’s mother, II3—proband’s paternal aunt, III1 - proband’s paternal grandfather, III2 - proband’s paternal grandmother, IV - proband’s paternal great-grandmother. **(B)** Limbs of family members, carrying the duplication 10q24.32. **(C)** Radiographs of the hands and feet of family members, carrying the duplication 10q24.32.

Since birth, the child has been under the care of an orthopedic specialist due to a congenital limb malformation. The right hand has fused fourth and fifth fingers from the base to the nail phalanges (Figures 1B,C I1). There is also hypoplasia of the first, second, and third rays. On the left hand, the fourth and fifth fingers are fused up to the level of the nail phalanges, and there is hypoplasia of the first, second, and third fingers. The feet are split, represented by the first and fifth rays, and there are unrestricted movements in the ankles.

The father of the proband has syndactyly of the second and third fingers of the hands and ectrodactyly of the feet (Figures 1B,C II1). Neither the proband nor his father had any other health problems. According to the parents, the other family members are healthy.

**FIGURE 2:**
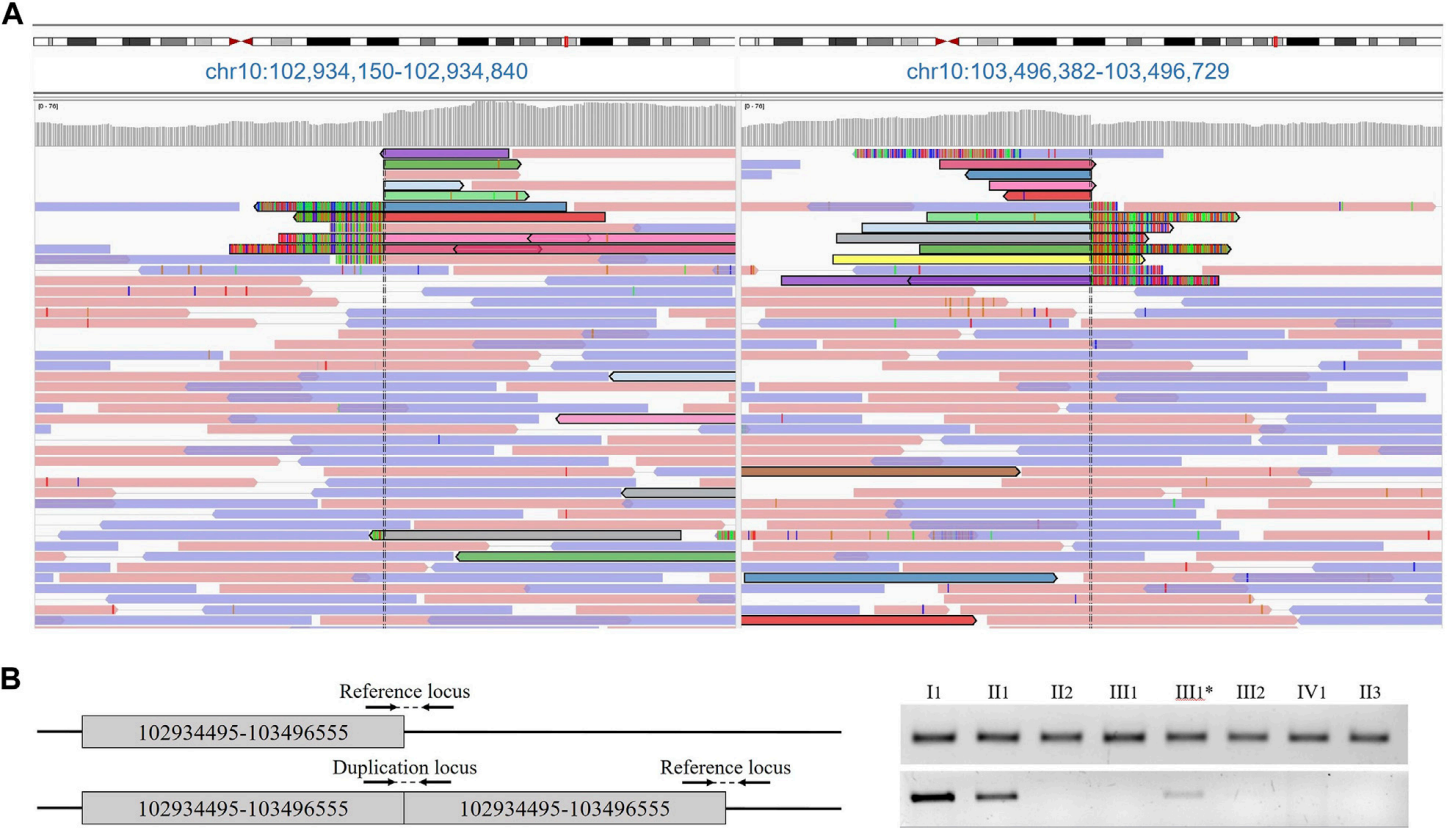
**(A)** Results of proband whole genome sequencing data. Paired tracks, indicated by different colors, define the boundaries of the duplication. **(B)** Primer arrangement for validation and duplication segregation in the family. Agarose gel electropheresis of PCR products of amplified loci.

At the age of 6 months, the proband was consulted by a medical geneticist and referred for whole-genome sequencing. By using whole-genome sequencing, a search for pathogenic variants associated with congenital anomalies of the hands and feet, as well as other hereditary diseases with similar phenotypic manifestations, was conducted in the patient. The study was performed on a DNA sample extracted from the proband’s peripheral blood. As a result of the whole-genome sequencing, a tandem duplication of a segment of chromosome 10 was identified with breakpoints at positions 102934495–103496555 bp, encompassing the *BTRC*, *POLL*, *FBXW4* and *LBX1* genes (Figure 2A). The size of the detected duplication is 562 kb.

The presence of the tandem duplication chr10:g.102934495_103496555dup was confirmed in a heterozygous state in the DNA samples from peripheral blood of the patient and his father (Figure 2B). Additionally, we found that the paternal grandmother is also a carrier of the duplication in a mosaic form at a level of 10%. Despite the visual examination suggesting the presence of mild brachydactyly, radiological analysis did not reveal any signs of pathology in the grandmother (Figures 1B,C III1). The studied duplication was not detected in the other examined family members.

To confirm the mosaic nature of the inheritance of the duplication in the proband’s grandmother, we performed PCR analysis on DNA samples extracted from buccal epithelial cells. However, the investigated structural variant was not detected.

## 4 Discussion

The SHFM group of disorders is characterized by a wide range of clinical manifestations and varying degrees of severity. This condition can appear as an isolated form, showing split hands and/or feet and syndactyly as the only features, or it may present along with diverse clinical and craniofacial characteristics. The associated findings can vary significantly among affected individuals and may include abnormalities in the craniofacial region, limbs, skeleton, skin, as well as hearing loss, microphthalmia, renal anomalies, developmental delay, and others (Umair and Hayat, 2020). Each individual case can present a distinct combination of these features, highlighting the diverse nature of this genetic disorder. Even in cases of isolated SHFM forms, the degree of phenotypic expression varies widely. Families have been described in which carriers of the same genetic variant exhibit different clinical manifestations (Dai et al., 2013; Holder-Espinasse et al., 2019). The clinical heterogeneity makes it challenging to determine a specific disease type and establish a diagnosis solely based on the clinical presentation. The classification of SHFM is primarily based on genetic variants that determine the underlying pathogenesis.

Depending on the chromosomal defects and genes involved, SHFM is divided into six types. For some of these types, specific genes and molecular mechanisms responsible for the clinical presentation have been identified, while for others, only the chromosomal locus is known to be rearranged, leading to the development of the pathological phenotype.

SHFM type 1 maps to 7q21.2-q21.3, with candidate genes including *DLX5, DLX6*, and *DSS1*. SHFM type 2 maps to Xq26, and the candidate gene remains unidentified. SHFM type 3 is caused by duplication involving *BTRC* and *POLL* at 10q24.3. SHFM type 4 is due to mutations in the *P63* gene at 3q27. The cause of SHFM type 5 is considered to be the deletion of the chromosomal region 2q31, which contains genes *DLX1* and *DLX2* associated with this condition. Mutations in the *WNT10B* gene, located on chromosome 12q13, have been found to be responsible for the occurrence of SHFM type 6 (Sowinska-Seidler et al., 2014). The search for candidate genes is ongoing at the present time, leading to the discovery and description of new types of SHFM (Spielmann et al., 2016; Umair et al., 2018; Papasozomenou et al., 2019; Funk et al., 2020; Elsner et al., 2021; Schnur et al., 2021; Parveen et al., 2023).

Depending on the suspected disease type by the physician, several main methods of molecular diagnostics are used (Umair and Hayat, 2020). In cases where a specific gene is known, and pathogenic variants within it lead to the development of this type of disease, the most straightforward and effective molecular diagnostic method is Sanger sequencing (Ullah et al., 2017; Umair and Hayat, 2020).

At this time the predominant molecular diagnostic approach for identifying different types of SHFM associated with structural chromosomal rearrangements is microarray-based comparative genomic hybridization (aCGH) (Dimitrov et al., 2010; Holder-Espinasse et al., 2019). However, chromosomal microarray analysis does not provide information about the precise boundaries of chromosomal rearrangements, and therefore, this data is insufficient for conducting variant segregation analysis within the family.

In this study, we dealt with a family with variable phenotypic features: the proband has fully hypoplastic 1, 2, 3 fingers on both hands, while the father has syndactyly of the second and third fingers of the hands. The feet of the proband are split and represented by 1 and 5 rays, while the father has ectrodactyly of the feet. Due to this clinical heterogeneity and the inability to confidently assume a specific type of SHFM, we chose the method of whole-genome sequencing for molecular diagnostics. Despite the fact that deep sequencing is a much more time-consuming and expensive method, it allows for the detection of both point mutations, typical for types 1, 4, and 6, as well as precisely determining the boundaries of structural rearrangements characteristic of types 2, 3, and 5 SHFM. Thus, it covers all possible genetic variants responsible for the development of the pathology.

As a result, it was discovered that, despite differing clinical presentations, both the proband and his father carry the same tandem duplication situated on the long arm of chromosome 10, involving the *BTRC*, *POLL*, *FBXW4* and *LBX* genes. Furthermore, segregation analysis revealed the presence of the above-mentioned duplication in the grandmother’s blood sample, albeit in a mosaic state. Despite the fact that X-ray examination of the grandmother’s hands and feet did not reveal significant phalangeal shortening, brachydactyly of the hands is visually evident upon inspection (Figure 1). Similar cases have been described twice before: in both instances, siblings with SHFM type 3 inherited the pathological rearrangement from healthy mothers who were mosaic carriers of the duplication (Dimitrov et al., 2010; Filho et al., 2011).

In mice with syndactyly, which serves as a model of SHFM type 3, it has been shown that the influence of epigenetic factors can cause incomplete penetrance and, consequently, determine the severity of the patient’s phenotype (Kano et al., 2007). This may be the reason for the clinical difference between the father and the proband.

Duplications of this locus have been described in patients with type 3 SHFM (Umair and Hayat, 2020). The size of the duplicated locus in the family we described is 562 kb, which corresponds to the data from previous studies where duplication sizes ranged from 120 kb to 658 kb (Sowinska-Seidler et al., 2014; Li et al., 2015).

To date, only a few dozen patients with a molecularly diagnosed SHFM type 3 have been described. Our research contributes to the understanding of the pathogenesis underlying the disease and its phenotypic manifestations. Furthermore, the accumulation of a sufficient amount of data may, in the future, help determine the cause of the clinical heterogeneity of the condition.

## 5 Conclusion

In our current research, we used whole-genome sequencing to detect tandem duplication chr10:g.102934495_103496555dup in three individuals from the same family. Among them, two individuals are afflicted by SHFM type 3, while one carries the variant in a mosaic state without showing any symptoms. Furthermore, we have observed different levels of disease severity within this family. We believe that this case study adds new knowledge regarding the molecular causes of this condition.

## Data Availability

The datasets presented in this article are not readily available because patient’s personal data is not disclosed. Requests to access the datasets should be directed to DA, mkda6ka@mail.ru.

## References

[B1] Auerbach C. (1956). A possible case of delayed mutation in man. Ann. Hum. Genet. 20, 266–269. 10.1111/j.1469-1809.1955.tb01281.x 13314396

[B2] Buttiens M. Fryns J. P. (1987). Apparently new autosomal recessive syndrome of mental retardation, distal limb deficiencies, oral involvement, and possible renal defect. Am. J. Med. Genet. 27, 651–660. 10.1002/ajmg.1320270319 3631136

[B3] Cova G. Glaser J. Schopflin R. Prada-Medina C. A. Ali S. Franke M. (2023). Combinatorial effects on gene expression at the Lbx1/Fgf8 locus resolve split-hand/foot malformation type 3. Nat. Commun. 14, 1475. 10.1038/s41467-023-37057-z 36928426 PMC10020157

[B4] Dai L. Deng Y. Li N. Xie L. Mao M. Zhu J. (2013). Discontinuous microduplications at chromosome 10q24.31 identified in a Chinese family with split hand and foot malformation. BMC Med. Genet. 14, 45. 10.1186/1471-2350-14-45 23596994 PMC3637097

[B5] De Smet L. Devriendt K. Fryns J. P. (2001). Further evidence for germinal mosaicism in cleft hand/cleft foot syndrome. Two affected halfsisters and normal father. Genet. Couns. 12, 251–254. 11693788

[B6] Dimitrov B. I. De Ravel T. Van Driessche J. De Die-Smulders C. Toutain A. Vermeesch J. R. (2010). Distal limb deficiencies, micrognathia syndrome, and syndromic forms of split hand foot malformation (SHFM) are caused by chromosome 10q genomic rearrangements. J. Med. Genet. 47, 103–111. 10.1136/jmg.2008.065888 19584065

[B7] Duijf P. H. Van Bokhoven H. Brunner H. G. (2003). Pathogenesis of split-hand/split-foot malformation. *Hum. Mol. Genet*. 12 Spec. No 1, R51–R60. 10.1093/hmg/ddg090 12668597

[B8] Elliott A. M. Evans J. A. (2006). Genotype-phenotype correlations in mapped split hand foot malformation (SHFM) patients. Am. J. Med. Genet. A 140, 1419–1427. 10.1002/ajmg.a.31244 16688749

[B9] Elliott A. M. Evans J. A. Chudley A. E. (2005). Split hand foot malformation (SHFM). Clin. Genet. 68, 501–505. 10.1111/j.1399-0004.2005.00530.x 16283879

[B10] Elsner J. Mensah M. A. Holtgrewe M. Hertzberg J. Bigoni S. Busche A. (2021). Genome sequencing in families with congenital limb malformations. Hum. Genet. 140, 1229–1239. 10.1007/s00439-021-02295-y 34159400 PMC8263393

[B11] Filho A. B. Souza J. Faucz F. R. Sotomaior V. S. Dupont B. Bartel F. (2011). Somatic/gonadal mosaicism in a syndromic form of ectrodactyly, including eye abnormalities, documented through array-based comparative genomic hybridization. Am. J. Med. Genet. A 155A, 1152–1156. 10.1002/ajmg.a.33942 21485001

[B12] Funk C. R. Huey E. S. May M. M. Peng Y. Michonova E. Best R. G. (2020). Rare missense variant p.Ala505Ser in the ZAK protein observed in a patient with split-hand/foot malformation from a non-consanguineous pedigree. J. Int. Med. Res. 48, 300060519879293. 10.1177/0300060519879293 32266845 PMC7144677

[B13] Guero S. Holder-Espinasse M. (2019). Insights into the pathogenesis and treatment of split/hand foot malformation (cleft hand/foot). J. Hand Surg. Eur. 44, 80–87. 10.1177/1753193418807375 30380990

[B14] Holder-Espinasse M. Jamsheer A. Escande F. Andrieux J. Petit F. Sowinska-Seidler A. (2019). Duplication of 10q24 locus: broadening the clinical and radiological spectrum. Eur. J. Hum. Genet. 27, 525–534. 10.1038/s41431-018-0326-9 30622331 PMC6460637

[B15] Kano H. Kurahashi H. Toda T. (2007). Genetically regulated epigenetic transcriptional activation of retrotransposon insertion confers mouse dactylaplasia phenotype. Proc. Natl. Acad. Sci. U. S. A. 104, 19034–19039. 10.1073/pnas.0705483104 17984064 PMC2141903

[B16] Lai S. Zhang X. Feng L. He M. Wang S. (2020). The prenatal diagnosis and genetic counseling of chromosomal micro-duplication on 10q24.3 in a fetus: a case report and a brief review of the literature. Med. Baltim. 99, e22533. 10.1097/MD.0000000000022533 PMC7571886 33080687

[B17] Li C. F. Angione K. Milunsky J. M. (2015). Identification of critical region responsible for split hand/foot malformation type 3 (SHFM3) phenotype through systematic review of literature and mapping of breakpoints using microarray data. Microarrays (Basel) 5, 2. 10.3390/microarrays5010002 27600068 PMC5003447

[B18] Papasozomenou P. Papoulidis I. Mikos T. Zafrakas M. (2019). Split hand foot malformation syndrome: a novel heterozygous FGFR1 mutation detected by next generation sequencing. Curr. Genomics 20, 226–230. 10.2174/1389202920666190530092856 31929729 PMC6935954

[B19] Parveen A. Tariq M. Khan S. A. Kakar N. Arif A. Wasif N. (2023). A novel frameshift variant in UBA2 causing split-hand/foot malformations in a Pakistani family. Hum. Genome Var. 10, 16. 10.1038/s41439-023-00242-z 37221169 PMC10206101

[B20] Qiu L. Li C. Zheng G. Yang T. Yang F. (2022). Microduplication of BTRC detected in a Chinese family with split hand/foot malformation type 3. Clin. Genet. 102, 451–456. 10.1111/cge.14204 35908152

[B21] Rath A. (2022). Prevalence of rare diseases: bibliographic data. Orphanet Rep. Ser. 1.

[B22] Schnur R. E. Yousaf S. Liu J. Chung W. K. Rhodes L. Marble M. (2021). UBA2 variants underlie a recognizable syndrome with variable aplasia cutis congenita and ectrodactyly. Genet. Med. 23, 1624–1635. 10.1038/s41436-021-01182-1 34040189 PMC8463496

[B23] Sowinska-Seidler A. Socha M. Jamsheer A. (2014). Split-hand/foot malformation - molecular cause and implications in genetic counseling. J. Appl. Genet. 55, 105–115. 10.1007/s13353-013-0178-5 24163146 PMC3909621

[B24] Spielmann M. Kakar N. Tayebi N. Leettola C. Nurnberg G. Sowada N. (2016). Exome sequencing and CRISPR/Cas genome editing identify mutations of ZAK as a cause of limb defects in humans and mice. Genome Res. 26, 183–191. 10.1101/gr.199430.115 26755636 PMC4728371

[B25] Ullah A. Hammid A. Umair M. Ahmad W. (2017). A novel heterozygous intragenic sequence variant in DLX6 probably underlies first case of autosomal dominant split-hand/foot malformation type 1. Mol. Syndromol. 8, 79–84. 10.1159/000453350 28611547 PMC5465671

[B26] Umair M. Hayat A. (2020). Nonsyndromic split-hand/foot malformation: recent classification. Mol. Syndromol. 10, 243–254. 10.1159/000502784 32021595 PMC6997797

[B27] Umair M. Ullah A. Abbas S. Ahmad F. Basit S. Ahmad W. (2018). First direct evidence of involvement of a homozygous loss-of-function variant in the EPS15L1 gene underlying split-hand/split-foot malformation. Clin. Genet. 93, 699–702. 10.1111/cge.13152 29023680

